# Pregabalin as a Potential Adjunct in the Management of Pruritus in Prurigo Nodularis: A Case Report

**DOI:** 10.7759/cureus.88297

**Published:** 2025-07-19

**Authors:** Yoshihito Mima, Masako Yamamoto, Ken Iozumi

**Affiliations:** 1 Department of Dermatology, Tokyo Metropolitan Police Hospital, Tokyo, JPN

**Keywords:** cgrp, gaba, nemolizumab, pregabalin, substance p, vgcc α2δ

## Abstract

Prurigo nodularis (PN) is a chronic inflammatory skin disorder characterized by intense pruritus and hyperkeratotic nodules. Pregabalin, a γ-aminobutyric acid (GABA) analogue commonly used for neuropathic pain, has shown promise in alleviating chronic pruritus, including PN. Although not currently approved for the treatment of PN or chronic pruritus, pregabalin is considered a promising off-label therapeutic option. We report the case of a 69-year-old woman with PN who achieved complete resolution of skin lesions and marked improvement in pruritus following treatment with nemolizumab. However, after discontinuation of pregabalin, which had been prescribed for coexisting sciatic pain, the patient experienced a recurrence of pruritus over the course of two months, despite the continued absence of visible skin lesions. Reintroduction of low-dose pregabalin led to symptom stabilization within two months, suggesting that pregabalin may have contributed to the control of pruritus. Pregabalin may exert antipruritic effects by inhibiting the α2δ subunit of voltage-gated calcium channels and suppressing the release of excitatory neurotransmitters such as substance P and calcitonin gene-related peptide. This case highlights the potential utility of pregabalin in managing PN-associated pruritus, particularly in cases with a suspected neuropathic itch component. Further clinical studies are warranted to clarify the efficacy of pregabalin and better define its role as an adjunctive treatment for chronic pruritus in PN.

## Introduction

Prurigo nodularis (PN) is a chronic inflammatory skin disease characterized by intensely pruritic papules, pustules, or nodules ranging in size from a few millimeters to 2-3 cm. These lesions typically exhibit a symmetrical distribution on the limbs and trunk [1]. Similar to atopic dermatitis (AD), PN is maintained by the “itch-scratch cycle” and involves both immune and neural dysregulation in its pathogenesis. Known risk factors include eczema, psychiatric disorders, malignancies, hepatic or renal dysfunction, diabetes mellitus, and HIV infection [2]. Histopathologically, PN lesions are marked by Th2-skewed inflammatory cell infiltration, including eosinophils, macrophages, and T cells. Like in AD, Th2 cytokines, such as IL-4, IL-13, and IL-31, activate peripheral nerve TRP channels, thereby triggering itch [1-3]. Additional mediators, such as tryptase, histamine, prostaglandins, and neuropeptides, are also implicated in the pruritic process [4]. Neurological changes, including increased nerve fiber density and elevated expression of calcitonin gene-related peptide (CGRP) and substance P, are believed to further intensify itch [4]. Notably, the expression levels of IL-31 and oncostatin M (OSM) are higher in PN lesions than in AD. These cytokines signal through a shared receptor complex composed of IL-31 receptor A and OSM receptor β (OSMRβ), directly activating itch-transmitting nerves and promoting nerve growth and immune cell activation [5]. In addition to topical corticosteroids, other conventional treatments such as calcineurin inhibitors, phototherapy, immunosuppressants, anticonvulsants, and antidepressants have received attention. More recently, biologic therapies targeting IL-4, IL-13, and IL-31 have emerged as promising new treatment options [6]. Furthermore, although not officially approved for this indication, pregabalin has shown therapeutic potential in PN and chronic pruritus [7,8].

Pregabalin is a γ-aminobutyric acid (GABA) analogue classified as an antiepileptic and neuropathic pain medication that acts on the central nervous system. Despite its structural similarity to GABA, pregabalin does not bind to GABA receptors. Instead, it binds to the α2δ subunit of voltage-gated calcium channels (VGCCs), thereby reducing calcium influx and inhibiting the presynaptic release of excitatory neurotransmitters such as glutamate, substance P, and norepinephrine [9]. This mechanism helps suppress neuronal hyperexcitability, contributing to pain and seizure control. By stabilizing neuronal activity, pregabalin may indirectly promote GABA-related inhibitory transmission [10].

Clinical reports have described cases of patients with PN who experienced marked improvement in pruritus following pregabalin administration for unrelated neuropathic pain. In a clinical study involving 30 patients with PN, 76% demonstrated favorable responses in terms of both itch and lesion improvement following pregabalin treatment, supporting its off-label utility in PN [7,8].

Herein, we report a case of a patient with PN who experienced a relapse of pruritus following dose reduction of pregabalin, which had been prescribed for sciatic pain, despite ongoing treatment with nemolizumab. To date, there have been few reports on the effects of pregabalin on PN-associated pruritus. This observation provides important insight, suggesting that pregabalin may have contributed to the control of pruritus in PN.

## Case presentation

**Figure 1:**
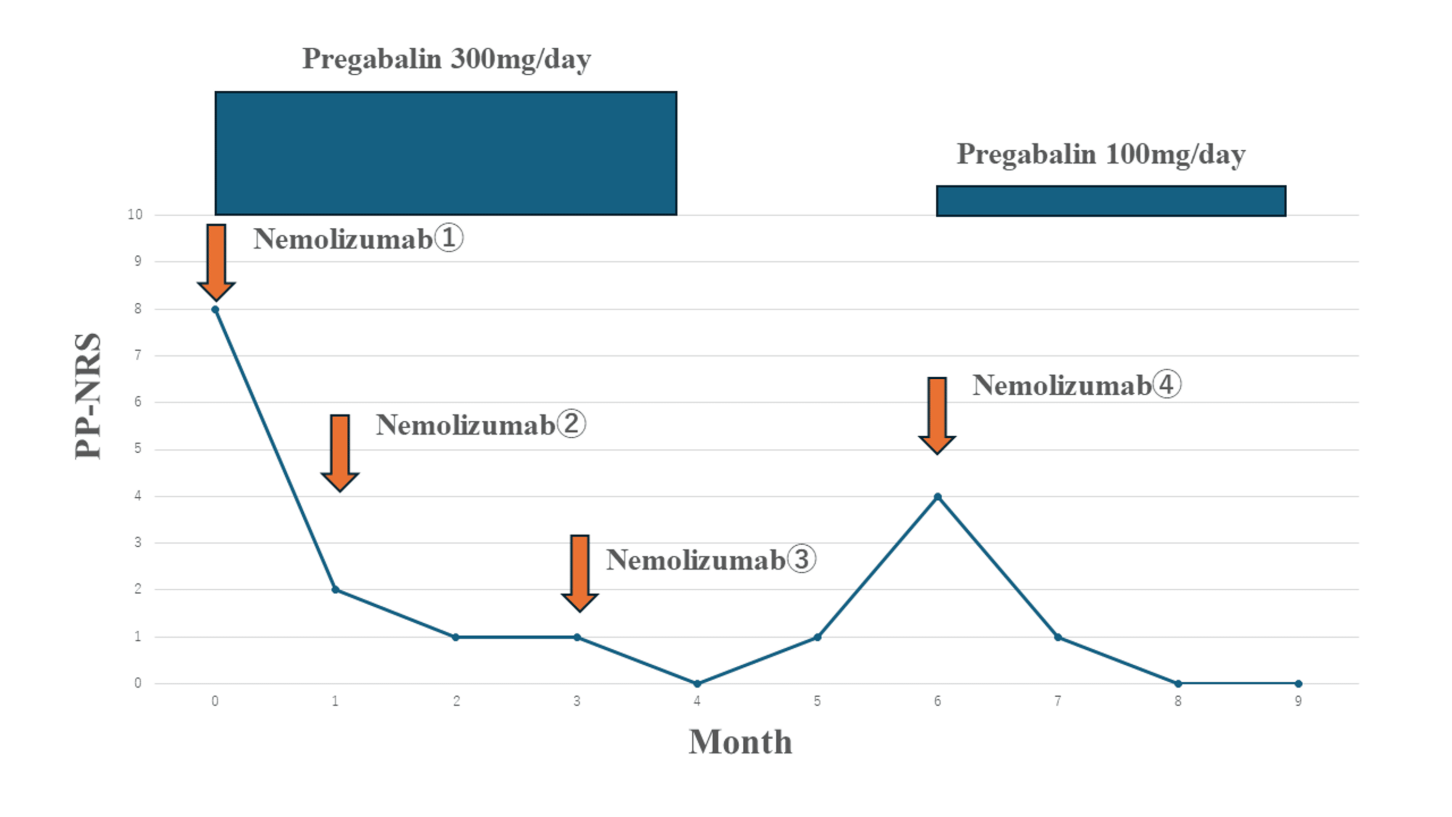
A timeline of the treatment course, including administration of nemolizumab and pregabalin. PP-NRS: Peak Pruritus Numerical Rating Scale

A 69-year-old woman with a history of hypertension and Hashimoto’s thyroiditis presented with multiple pruritic nodules on her back and was referred to our department with a diagnosis of PN. At the initial visit, her Peak Pruritus Numerical Rating Scale (PP-NRS) score was eight, and the Prurigo Nodularis Investigator’s Global Assessment (PN-IGA) score was three. Despite years of topical corticosteroid treatment, both pruritus and skin lesions remained refractory. Consequently, nemolizumab therapy was initiated with an initial dose of 60 mg, followed by 30 mg at intervals of more than one month. As her pruritus gradually improved, the dosing interval was extended to every two months (Figure 1).

**Figure 2:**
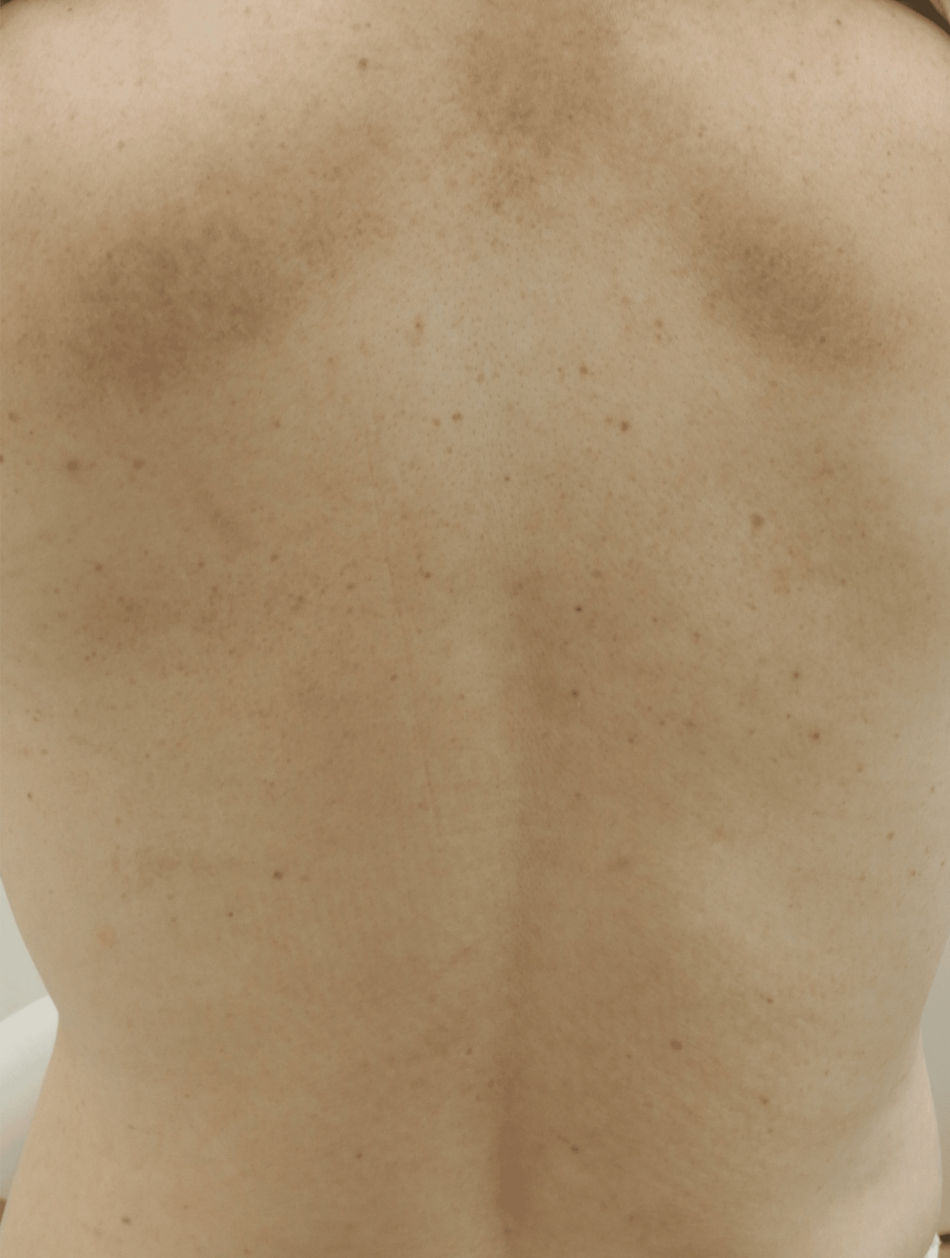
All pruriginous nodules on the back had flattened, with post-inflammatory hyperpigmentation nearly resolved.

After the fourth dose, she achieved a PN-IGA score of zero, and all skin lesions resolved (Figure 2).

However, pregabalin at a dose of 300 mg/day, prescribed by another department for sciatic pain, was continued through the third dose of nemolizumab. With the combined effect of pregabalin, pruritus improved markedly: the PP-NRS score decreased from eight to two after the first dose of nemolizumab, from two to one after the second dose, and from one to zero after the third dose, indicating effective pruritus control. However, one month after the third dose, pregabalin was discontinued, leading to a worsening of pruritus, with the PP-NRS increasing to four. In response, pregabalin was reintroduced at 100 mg/day concurrently with the fourth dose of nemolizumab, resulting in improvement. Currently, three months after restarting pregabalin at 100 mg/day, the patient’s pruritus remains well-controlled with a PP-NRS score of zero. Throughout the treatment period, in addition to pregabalin and nemolizumab, moisturizers and topical corticosteroids were used as needed during episodes of severe pruritus.

## Discussion

In a patient undergoing nemolizumab therapy for PN, discontinuation of pregabalin led to a relapse of pruritus, which subsequently improved following re-initiation of low-dose pregabalin. In our case, the patient’s pruritus was unresponsive to previous antihistamines, moisturizers, topical corticosteroids, and nemolizumab, but improved with pregabalin, suggesting a possible involvement of neuropathic pruritus. As pregabalin was reintroduced alongside nemolizumab, it is likely that nemolizumab also contributed to the improvement in pruritus to some extent. However, a comparison of pruritus levels following the third and fourth doses of nemolizumab, with and without pregabalin, suggests that pregabalin may have played a significant role in pruritus control. Pregabalin has been reported to improve not only pruritus but also quality of life (QOL) among patients with chronic pruritus [11]. Consistent with these findings, our patient also experienced improved sleep dysfunction and overall QOL following pregabalin therapy.

Itch and pain share common neural pathways; both are transmitted via C-fibers in the dorsal horn of the spinal cord and ascend through the lateral spinothalamic tract to the thalamus and somatosensory cortex. Given this overlap, pregabalin, originally developed as an analgesic, may also exert antipruritic effects [12,13]. Structurally similar to GABA, pregabalin does not act directly on GABA receptors. Instead, it exhibits high affinity for the α2δ subunit of VGCCs and interacts with N-methyl-D-aspartate (NMDA) receptor complexes, suppressing the release of excitatory neurotransmitters such as dopamine, serotonin, and norepinephrine. Through these mechanisms, pregabalin may increase the activation threshold of sensory neurons, inhibit calcium influx and glutamate release, and reduce synaptic transmission involved in itch signaling [14,15]. Studies using mouse models have shown that pregabalin suppresses the expression of the α2δ-1 subunit in the dorsal root ganglia, supporting its antipruritic effects [16,17]. This subunit is upregulated at sites of peripheral nerve injury, suggesting a link between pregabalin’s analgesic and antipruritic actions [16,17].

Chronic pruritus, including PN, is often multifactorial, involving skin barrier disruption, immune dysregulation, and neural alterations. Neuropathic itch is believed to arise from hypersensitization of either the peripheral or central nervous system. In PN, the itch-scratch cycle is thought to initiate keratinocyte activation and cytokine release, which further stimulates itch-sensitive neurons. This leads to sustained release of neuropeptides such as substance P and CGRP, promoting neurogenic inflammation. These neuropeptides contribute to vasodilation and mast cell degranulation, exacerbating chronic itch. Elevated levels of substance P have been reported in patients with chronic pruritus, suggesting ongoing sensory neuron stimulation [18,19].

Gabapentinoids, such as pregabalin, have been shown in animal models to suppress substance P and CGRP release from inflamed spinal tissues, indicating that their antipruritic effects may be mediated through modulation of these neuropeptides [14,17]. Thus, pregabalin may alleviate neuropathic itch by increasing the excitability threshold of sensory neurons via VGCC α2δ subunit inhibition and suppressing substance P and CGRP release.

Although pregabalin is currently approved only for neuropathic pain, its potential benefit in refractory pruritus warrants consideration. For patients with intractable itch not adequately controlled by conventional treatments, pregabalin may represent a viable therapeutic option. The limitations of this case include the fact that it is based on the clinical course of a single patient and the absence of a standardized pruritus assessment beyond the PP-NRS. Further accumulation of clinical evidence is needed to validate its efficacy in chronic pruritus.

## Conclusions

This case report describes a 69-year-old woman with PN who had been prescribed pregabalin for sciatic pain. Her pruritus initially improved with nemolizumab but recurred following the discontinuation of pregabalin. Reintroduction of low-dose pregabalin led to stabilization of symptoms, and continued use of pregabalin after discontinuation of nemolizumab further supported its potential role in itch control. Although pregabalin is not approved for the treatment of PN, it may alleviate pruritus by modulating calcium channels and suppressing the release of neuropeptides. This case highlights the potential utility of pregabalin as an adjunctive therapy in PN, particularly in cases where neuropathic itch is suspected. Further studies are warranted to validate its efficacy.
